# Risk factors for pregnancy related complications among urban slum and non-slum women in Bangladesh

**DOI:** 10.1186/s12884-019-2392-6

**Published:** 2019-07-08

**Authors:** Mirajul Islam, Nasrin Sultana

**Affiliations:** Department of Statistics, University of Dhaka, Dhaka, 1000 Bangladesh

**Keywords:** Pregnancy related complications, Maternal death, Mixed logistic regression model, UHS 2013, Urban areas in Bangladesh

## Abstract

**Background:**

Bangladesh is facing a higher maternal mortality and morbidity than many other developing countries in the world. The majority of these maternal deaths occur due to pregnancy related complications. Although health facilities in urban areas in Bangladesh are widely available, women living in underprivileged urban areas are least likely to receive the maternal health services and as a result, they face more pregnancy related complications. Unfortunately, there are only a few studies on complications during maternal and delivery period in these areas. We aim to investigate the factors responsible for pregnancy related complications in urban slum and non-slum areas.

**Methods:**

Data from the Urban Health Survey (UHS), 2013 were analyzed applying mixed logistic regression model. The response variable was complications during pregnancy, during/after delivery at the last birth and the total sample size was 6137. The adjusted odds ratios (AORs) along with their 95% confidence intervals (CIs) were also calculated to compare the magnitude of different risk factors for the pregnancy related complications.

**Results:**

Younger mothers (age < 18 years) at the birth of their children had 24% (OR = 1.24, 95% CI: 1.01, 1.54) more odds to experience complications during pregnancy/delivery or after delivery compared to older mothers aged 18 to 35 years. The increased risk of complications was found among primiparous women. Women living in urban slum areas had higher pregnancy related complications than women living elsewhere. Migrant mothers faced more complications than women-who were not migrants. Women had greater pregnancy related complications when they delivered boy child than girl child, presumably from an increased size of the baby and resultant birth obstruction, assisted delivery and post partum haemorrhage. Moreover, a wanted pregnancy had fewer significant complications during pregnancy/delivery or after delivery than an unwanted pregnancy.

**Conclusions:**

The study associates early maternal age, primiparity, unwanted pregnancy, women living in slum areas, women migrating from other cities or non-urban areas and NGO membership with increased risk of pregnancy related complications among urban women in Bangladesh. It is likely that addressing these risk factors for complications to the policymakers may help to reduce the maternal mortality and morbidity in Bangladesh.

## Background

Bangladesh, as are many other developing countries, is facing a rapid urbanization challenge as part of a global trend. About 67% of urban population growth is caused due to migration from non urban areas and many demographers expect that much of the future population growth will mainly occur in urban areas [1]. About 3.8% of the total national populations or, over 5.7 million people are living in urban slums with a high population density and poorer health condition [2]. Despite the ready availability of health services in most urban areas of Bangladesh, the disadvantaged populations in some urban areas have less access to quality health care compared with their counterparts in wealthier areas. Recent work has demonstrated that a public health clinic was available for only 7.3% of slums in Bangladesh [3] and the uneducated and poorest women in urban slums were less likely to receive maternal health care services from medically trained providers [4]. As a result, women in these disadvantaged populations may face relatively more pregnancy related complications and child birth period with an increased maternal deaths as well.

Annually, the number of women globally suffering from complications during/after pregnancy is approximately 9.5 million and more than 300,000 pregnant women will die [5, 6]. Millennium Development Goals (MDG) are targeted to reduce the maternal mortality rate (MMR) to 143 per 100,000 live births by 2015. This has not been achieved in Bangladesh as the MMR of this country was 176 per 100,000 live births in 2015, and despite making progress towards the reduction of MMR, it still has a higher MMR than many other countries [7]. However, this pace of reduction needs to improve with a view to meeting the challenge of the 2030 Sustainable Development Goal (SDG-3.1) of an MMR of less than 70 per 100,000 [8]. Therefore, understanding the causes of maternal deaths in Bangladesh is a primary requirement for further reduction in MMR.

About three fourths of maternal deaths are caused due to the complications during pregnancy and child birth period [9]. Haemorrhage, excessive vomiting, convulsion/fits, and oedema face/feet/body were considered as the high risk pregnancy related complications [10]. Moreover, heavy bleeding, prolonged labor, high fever or discharge, and convulsions were selected as pregnancy related complications in a study on health care seeking behavior [11]. A study conducted in Dhaka slums found that postpartum hemorrhage and eclampsia were the main causes of maternal deaths [12, 13]. The contribution of these major complications to high MMR deserves attention.

Since newborn health is closely linked with maternal health, reduction in complications during pregnancy should also reduce the high MMR, IMR and under-5 mortality rate. Unluckily, there are few studies on complications during/after pregnancy or after delivery in urban, and the slum areas of Bangladesh. Therefore, it is timely to focus on the risk factors of pregnancy and childbirth related complications so that strategies can be developed to reduce maternal mortality and morbidity as well as child mortality to achieve the SDGs.

This study aims to quantify complications during pregnancy/delivery or after delivery of women in urban areas of Bangladesh using Bangladesh Urban Health Survey (UHS), 2013 data. Since the data were collected using different clusters, the mixed logistic regression model was applied to take into account the random effect in the analysis due to these clusters in the data.

## Methods

### Data source

This was a secondary data analysis from the Bangladesh Urban Health Survey (UHS), 2013. The main objective of this survey was to provide detailed information on key health outcomes and service utilization indicators in slums and non-slums areas of City Corporations, and other urban areas in Bangladesh. Fieldwork for this survey started on July 23, 2013 and ended on December 12, 2013. Five types of questionnaires were used in the 2013 UHS survey: a men’s questionnaire, a community questionnaire, a women’s questionnaire, a verbal autopsy questionnaire and a household questionnaire. The UHS, 2013 data were downloaded from the University of North Carolina website at https://dataverse.unc.edu .

### Sampling design

The main purpose of sampling design of UHS, 2013 was to find key representative indicators for slum and non-slum populations in the 9 city corporations; and district municipalities and large towns referred as other urban areas. The sample collected in this survey is nationally representative. Under-five mortality and percentage of birth deliveries in health facilities for all births in the last three years were used as the key indicators for determining the sample size. For estimating “under-five mortality”, the sample size for non-slums was estimated to be 27000 households which was expected to obtain an estimate within a margin of error of 4 per 1000 births (95% CI of 19–27) [1]. With similar precision, the sample sizes for other urban areas and slum areas were estimated to be about 11,040 and 15,750 households to obtain estimates within a margin of error of nine (95% CI of 41–59) and eight (95% CI of 55–71) per 1,000 births, respectively. Similarly, for estimating “Percentage of facility deliveries”, the sample sizes were 15750, 11040 and 9000 households within margin of errors 3.4, 5 and 6 percentage points for slums, other urban areas and non-slum areas, respectively.

The sampling frame of this survey was a complete list of urban Mohallas in the 9 city corporation and other areas from the 2011 census. The 9 city corporations were Dhaka metropolitan area, Khulna, Rajshahi, Chittagong, Barisal, Comilla, Rangpur, Narayangang and Sylhet. A 3-stage stratified sampling procedure was used for the collection of data of the UHS, 2013. There were two strata: city corporations and other urban areas. In the first stage, the numbers of randomly selected Mohallas in city corporations and other urban areas were 450 and 184, respectively. A mapping activity was used to separate slum and non-slum clusters in each Mohalla. At second stage, two slum clusters and one non-slum clusters were randomly selected from each Mohalla. In the case of other urban strata, two clusters were randomly selected from each selected Mohalla. In the last stage of selection, households of the selected clusters were randomly selected by using a household listing activity.

### Participants

The ever married women who lived in urban slums or non-slums were questioned about their last birth in the preceding three years of the UHS in order to minimize recall bias. This allowed for analysis of 6142 live births. The final sample size was 6137 after excluding the five women who had missing data regarding the number of ANC visits.

### Response variable

Women were considered as having complication [11] if they had experienced any of the following five problems during their last pregnancy, during or after delivery: convulsion/fits, severe/heavy bleeding, prolonged labor (> 12 h), high fever with smelly discharge, and oedema face/feet/body. Thus, the response variable, complications variable was coded as 1, if respondent had any of the five complications and 0, if she did not.

### Exposure variables

Based on the available literature review, the following socioeconomic, demographic and individual fertility variables were considered as exposure variables in our analysis: wealth index, education level of mothers, access to media, NGO membership, region, number of children ever born, pregnancy intention at last birth, mother’s age at last birth, multiple last birth, delivery by medically trained provider (MTP), sex of last child, place of residence, migration status, at least 4 ANC visits and place of delivery. A woman was considered as migrant if she came to urban area from other city or non-urban area. However, the variables “access to media” and “NGO member” were not directly obtained from the survey data. These two variables were created from the available information in the survey data as follows: a woman was considered as exposed to media if she usually read a newspaper or magazine/ listened to radio/watched TV and she was treated as an NGO member belonging to any of the following organizations: Grameen Bank, BRAC, BRDB, ASHA and PROSHIKA.

### Statistical analyses

The UHS, 2013 data were collected from 1718 clusters: 450 in slums, 900 in non-slums and 368 in other urban areas. This clustering might result in the correlated responses. To allow a wide variety of correlation pattern to be explicitly modeled, a generalized linear mixed model (GLMM) was applied. Chi-square test of association was used in bivariate analysis to determine whether there existed significant association between the outcome and exposure variables. In the regression analysis, the crude odds ratios (CORs) and adjusted odds ratios (AORs) were calculated by exponentiating the unadjusted and adjusted effects of the covariates obtained from simple and multiple mixed logistic regression models, respectively.

Suppose, *Y*_*ik*_ and *X*_*ik*_ are the outcome variable and *p* × 1 vector of exposure variables, respectively taken from the *k*^*th*^ (*k* = 1, 2, …, *n*_*i*_) individual of the *i*^*th*^ (*i* = 1, 2, …, *m*) cluster. Also assume that *β* = (*β*_1_, *β*_2_, …*β*_*p*_) is the *p* × 1 vector of regression parameters. Then the GLMM for the binary response, *Y*_*ik*_ can be written as

$$ {\eta}_{ik}=\mathit{\ln}\frac{\pi_{ik}}{1-{\pi}_{ik}}={x}_{ik}^{\prime}\beta +{v}_i $$

where *π*_*ik*_ = *E*(*Y*_*ik*_| *v*_*i*_) and *v*_*i*_ is the random intercept term that is assumed to follow normal distribution with mean 0 and variance ${\sigma}_v^2$. Therefore, the conditional log-likelihood function can be written as

$$ l\left(\beta, {\sigma}_v^2|{x}_{ik},{v}_i\right)=\sum \limits_{i=1}^m\sum \limits_{k=1}^{n_i}\ln \left(f\left({y}_{ik}|{x}_{ik},{v}_i\right)\right) $$

The estimates of the parameters *β* and ${\sigma}_v^2$ can be obtained by maximizing the following marginalized likelihood function

$$ L\left(\beta, {\sigma}_v^2\right)={\int}_{-\infty}^{\infty}\left\{\prod \limits_{i=1}^m\prod \limits_{k=1}^{n_i}f\left({y}_{ik}|{x}_{ik},{v}_i\right)\right\}d{v}_i. $$

The intra-cluster correlation coefficient, *ρ* can be measured by the variance component as [14] $\rho =\frac{\sigma_v^2}{\sigma_v^2+\frac{\pi^2}{3}}$.

## Results

Among 6137 ever married women included in this study, 21.5% had at least one of the five complications during their last pregnancy. It is observed that 18.4% of the respondents had no education whereas the highest percentage (36%) completed their secondary education. The economic status of the selected mothers was poor for 57.4%. The highest number of women (64.1%) was from Dhaka division. The least number of women was from the age group above 35 whereas the highest number was from the age group 18–35. Approximately 48% of the selected mothers had two or three children and 40.6% of them had the last child as first ever born children during three years preceding the survey. A vast majority of the slum mothers considered in this study had access to media. Only 11.3% of the respondents had NGO membership. Among the mothers selected in this study, 82% mothers wanted to be pregnant at the time of their last pregnancy. The percentages of respondents coming from city corporation slums and city corporation non-slums were 66.2 and 33.8, respectively. Almost all of the respondents selected in our study had single birth as their last birth. Among the last children, 50.6% children were boys and rest 49.4% were girls. Almost half of the deliveries of the last children occurred at home. It is also found that 66.6% of the respondents included in this study from slum and non-slum urban areas were migrants that is, they were not born in the same place where they lived at the time of survey.

The contingency tables with row percentages were constructed to determine the distribution of complications for each category of exposure variables. Chi-square test of association were performed to determine which selected variables had significant association with pregnancy related complications. These percentage distributions, and *p*-values obtained from chi-square tests are presented in Table 1. It is observed from the table that all the variables except economic status, access to media, migration status, place of residence, and education level of mothers had significant associations with complications of mothers during pregnancy, during delivery or after delivery from bivariate analysis. Moreover, the unadjusted effects of the covariates were also calculated by applying mixed logistic regression model through crude odds ratios (COR). These CORs along with their corresponding 95% CIs have been presented in Table 2.

**Table 1:** Biological, demographic and socioeconomic variables by pregnancy related complications

Covariates	Complications during pregnancy/ delivery or after delivery		*p*-value
	Yes-N(%)^r^	No-N(%)^r^	
Mothers’ age at birth			
< 18	180 (26.6)	496 (73.4)	0.001
18–35	1078 (20.7)	4131 (79.3)	
> 35	60 (23.8)	192 (76.2)	
Children ever born			
1	593 (23.8)	1901 (76.2)	0.001
2–3	591 (20.0)	2361 (80.0)	
> 3	134 (19.4)	557 (80.6)	
Multiple last birth			
Yes	18 (31.6)	39 (68.4)	0.062
No	1300 (21.4)	4780 (78.6)	
Wanted pregnancy			
Yes	1031 (20.5)	4001 (79.5)	< 0.001
No	287 (26.0)	818 (74.0)	
At least 4 ANC visits			
Yes	583 (24.0)	1849 (76.0)	< 0.001
No	735 (19.8)	2970 (80.2)	
Place of delivery			
Home	558 (17.6)	2614 (82.4)	< 0.001
Health facility	760 (25.6)	2205 (74.4)	
Delivery by MTP			
Yes	738 (25.5)	2151 (74.5)	< 0.001
No	580 (17.9)	2668 (82.1)	
Sex of last child			
Boy	710 (22.9)	2393 (77.1)	0.007
Girl	608 (20.0)	2426 (80.0)	
Region			
Dhaka	894 (22.7)	3041 (77.3)	< 0.001
Chittagong	230 (16.9)	1134 (83.1)	
Others	194 (23.2)	644 (76.8)	
Place of residence			
Slum	888 (21.9)	3174 (78.1)	0.304
Non-Slum	430 (20.7)	1645 (79.3)	
Migration			
Migrant	900 (22.0)	3189 (78.0)	0.150
Non-migrant	418 (20.4)	1630 (79.6)	
Education level			
No education	230 (20.4)	899 (79.6)	0.227
Primary	429 (20.8)	1638 (79.2)	
Secondary/Higher	659 (22.4)	2282 (77.6)	
Access to media			
Yes	1160 (21.4)	4256 (78.6)	0.761
No	158 (21.9)	563 (78.1)	
NGO membership			
Yes	171 (24.7)	520 (75.3)	0.026
No	1147 (21.1)	4299 (78.9)	
Economic status			
Poor	758 (21.5)	2763 (78.5)	0.278
Middle	392 (20.6)	1509 (79.4)	
Rich	168 (23.5)	547 (76.5)	

^r^row percentage

**Table 2:** Crude odds ratios (CORs) and adjusted odds ratios (AORs) with 95% CIs obtained from mixed logistic regression model

Covariates	COR	95% CI of COR	AOR	95% CI of AOR
Mothers’ age at birth				
< 18	1.41	(0.22, 0.26)	1.24	(1.01, 1.54)
18–35	1	–	1	–
> 35	1.21	(0.84, 1.58)	1.32	(0.94, 1.84)
Children ever born				
1	1.25	(1.09, 1.42)	1.26	(1.08, 1.46)
2–3	1	–	1	–
> 3	0.96	(0.75, 1.17)	0.85	(0.67, 1.08)
Multiple last birth				
Yes	1.79	(0.74, 2.84)	1.91	(1.03, 3.44)
No	1	–	1	–
Wanted pregnancy				
Yes	0.73	(0.62, 0.85)	0.67	(0.56, 0.79)
No	1	–	1	–
At least 4 ANC visits				
Yes	1.31	(1.14, 1.48)	1.19	(1.03, 1.38)
No	1	–	1	–
Place of delivery				
Home	0.61	(0.53, 0.69)	0.71	(0.55, 0.91)
Health facility	1	–	1	–
Delivery by MTP				
Yes	1.61	(1.40, 1.82)	1.21	(0.94, 1.56)
No	1	–	1	–
Sex of last child				
Boy	1.18	(1.03, 1.33)	1.17	(1.03, 1.34)
Girl	1	–	1	–
Region				
Dhaka	0.69	(0.56, 0.81)	1	–
Chittagong	1	–	0.72	(0.60, 0.87)
Others	1.02	(0.81, 1.23)	0.94	(0.76, 1.17)
Place of residence				
Slum	1.07	(0.92, 1.23)	1.22	(1.05, 1.43)
Non-Slum	1	–	1	–
Migration				
Migrant	1.11	(0.95, 1.26)	1.21	(1.05, 1.40)
Non-migrant	1	–	1	–
Education level				
No education	1	–		
Primary	1.02	(0.83, 1.21)		
Secondary/Higher	1.15	(0.94, 1.35)		
Access to media				
Yes	0.97	(0.78, 1.16)		
No	1	–		
NGO membership				
Yes	1.23	(0.99, 1.47)	1.29	(1.05, 1.58)
No	1	–	1	–
Economic status				
Poor	1.07	(0.92, 1.23)		
Middle	1	–		
Rich	1.21	(0.95, 1.48)		
Constant			0.23	(0.16, 0.33)
-2loglikelihood			6209.8	
Variance component			0.218	

The variables that were found to have significant association with complications in bivariate analysis were further examined in a regression analysis for estimating the adjusted effects of these covariates. Although place of residence and migration status showed insignificant association with the pregnancy related complications, they were included in the regression model of finding adjusted effects so that the study can focus on slum and migrant women in the urban areas. The adjusted odds ratios obtained by performing mixed logistic regression in Table 2 have shown that women aged less than 18 years at last birth had 24% higher odds of pregnancy related complications than women aged 18 to 35 years at last birth (AOR = 1.24, 95% CI: 1.01, 1.54). Mothers who had one ever born child had 26% more odds to face complications during pregnancy, during/after delivery than mothers with two or three ever born children.

For multiple last birth, the odds of pregnancy related complications was 91% higher than single last birth. Furthermore, women who wanted to be pregnant at last birth had significantly less odds of complications compared to those who did not want (AOR = 0.67, 95% CI: 0.56, 0.79).

The regression results also showed that women who received at least 4 ANC visits during pregnancy had 19% higher odds of having pregnancy related complications than women not taking at least 4 ANC visits (AOR = 1.19, 95% CI: 1.03, 1.38). It was also shown that mothers who gave birth at home had 29% less odds to have reported complications before, during/after delivery than mothers giving birth at health facility (AOR = 0.71, 95% CI: 0.55, 0.91), presumably as they were referred to hospitals for these complications in the first place. The odds of complications among mothers with a boy as a last child was 17% higher than that among mothers with girl as a last child (AOR = 1.17, 95% CI: 1.03, 1.34).

Women living in Chittagong division had 28% less odds of complications than women in Dhaka division. Moreover, mothers from slum areas experienced greater complication than other mothers (AOR = 1.22, 95% CI: 1.05, 1.43). Not surprisingly, migration status was also found to be a significant factor for complications during pregnancy, during/after delivery. Migrant women had 21% more odds to face complications than non-migrant women. NGO members had higher odds of complications compared to women who were not NGO member. The estimate of variance component of the random effects was found to be 0.218. Intra-cluster correlation coefficient which was calculated from this estimate was found to be 0.062.

Both fixed logistic and mixed logistic regression models were applied to analyze the data used in this study and it was found that Akaike Information Criterion (AIC) value of the mixed model was 6243.8 whereas it was 6259.7 for fixed logistic model. That is, mixed model had smaller AIC value than fixed model. Therefore, mixed logistic regression model fits the data better than fixed logistic regression model.

## Discussions

The adjusted analysis conducted in this study revealed that child’s birth at early age of mother, primiparity, multiple birth, at least 4 ANC, a male child, a slum as a place of residence, migration, and NGO membership increased the chance of complications during pregnancy, during/after delivery among city corporation women in Bangladesh.

In Bangladesh, more than half of the women are married by their 18th birthday [15] and an earlier age at marriage is increasing teenage pregnancy. This study has shown that mothers giving birth at early age (< 18 years) face more pregnancy related complications. In addition, women at the birth of their first child suffer more from complications during pregnancy, during/after delivery.

Women with unwanted pregnancies also had an increased risk of facing pregnancy related complications and mothers faced more pregnancy complications at the deliveries of male child (perhaps from a larger size of baby with a resultant obstructed labour, operative delivery and PPH). Effective family planning and better maternal education should allow better access to contraception especially for the women who are at risk for unwanted pregnancy.

The regression analysis also found that women receiving at least four ANC visits and giving deliveries at health facilities had more complications than their counterparts. The reason for this might be that women in urban slum and non-slum areas usually did not take ANC visits and go to health facility for delivery unless they faced any problems during their pregnancy and half of the slum mothers received at least 4 ANC visits during pregnancy from non-medically trained providers [16]. Opposite results were found in India: women receiving no ANC during pregnancy had more pregnancy complications than women who took one or two ANC visits [17].

Women living in Chittagong division and urban non-slum areas had less pregnancy complications compared to Dhaka division and urban slum areas, respectively. The explanation for an increased number of pregnancy complications among slum mothers might be that the level of use of healthcare was observed to be much lower among slum dwellers due to limited access to healthcare than that among the urban non-slum population of Bangladesh [18]. Moreover, migration status of the mother was also a responsible factor for increasing complications during pregnancy, during/after delivery in urban areas of Bangladesh. Women who were classed as a migrant experienced more pregnancy related complications compared to native women. The possible reason might be that women who migrated to urban lagged in getting health care services [19, 20] and were disadvantaged by wealth index moderately than urban native women [21].

## Conclusions

Maternal death is a concerning issue in developing countries such as Bangladesh. The main cause of maternal mortality and morbidity in Bangladesh is from complications occurring during/after pregnancy and childbirth. This analysis of UHS, 2013 data has revealed that there are many biological, social and demographic factors that were associated with complications during/after pregnancy/delivery among urban mothers in Bangladesh. In any strategy to reduce maternal mortality, focus should be given to migrant mothers and slum mothers so that they can get sufficient access to health care services. Reduction of unwanted and teenage pregnancy can play a vital role for reducing pregnancy related complications and hence maternal deaths. More effective care during pregnancy/delivery is needed for mothers with a large baby (often a boy) child as well as multiple births. Appropriate care must be available for the women at their first birth and this can be done by education and ready access to antenatal, intrapartum and postnatal care from medically trained providers.

## Data Availability

The dataset analysed in this study are available at the Website of University of North Carolina, USA repository, [https://dataverse.unc.edu].

## Abbreviations

ANC: Antenatal care
IMR: Infant mortality rate
MMR: Maternal mortality rate
MTP: Medically trained provider
PNC: Postnatal care
SBA: Skilled birth attendant
SDG: Sustainable Development Goal
